# Heat Wave–Associated Vibriosis, Sweden and Finland, 2014

**DOI:** 10.3201/eid2207.151996

**Published:** 2016-07

**Authors:** Craig Baker-Austin, Joaquin A. Trinanes, Saara Salmenlinna, Margareta Löfdahl, Anja Siitonen, Nick G.H. Taylor, Jaime Martinez-Urtaza

**Affiliations:** Centre for Environment Fisheries and Aquaculture Science, Weymouth, UK (C. Baker-Austin, N.G.H. Taylor);; National Oceanic and Atmospheric Administration, Miami, Florida, USA (J.A. Trinanes);; University of Miami, Miami (J.A. Trinanes); Universidad de Santiago de Compostela, Santiago de Compostela, Spain (J.A. Trinanes);; National Institute for Health and Welfare, Helsinki, Finland (S. Salmenlinna, A. Siitonen);; Public Health Agency of Sweden, Stockholm, Sweden (M. Löfdahl);; University of Bath, Bath, UK (J. Martinez-Urtaza)

**Keywords:** Wound infections, vibrios, heat wave, Baltic Sea, bacteria, Sweden, Finland, northern Europe, Vibrio

## Abstract

During summer 2014, a total of 89 *Vibrio* infections were reported in Sweden and Finland, substantially more yearly infections than previously have been reported in northern Europe. Infections were spread across most coastal counties of Sweden and Finland, but unusually, numerous infections were reported in subarctic regions; cases were reported as far north as 65°N, ≈100 miles (160 km) from the Arctic Circle. Most infections were caused by non-O1/O139 *V. cholerae* (70 cases, corresponding to 77% of the total, all strains were negative for the cholera toxin gene). An extreme heat wave in northern Scandinavia during summer 2014 led to unprecedented high sea surface temperatures, which appear to have been responsible for the emergence of *Vibrio* bacteria at these latitudes. The emergence of vibriosis in high-latitude regions requires improved diagnostic detection and clinical awareness of these emerging pathogens.

*Vibrio* species are among the most common gram-negative bacteria that inhabit surface waters throughout the world and are responsible for several severe infections in humans and animals (*1*). Infection usually begins after exposure to seawater or ingestion of raw or undercooked seafood (*2*,*3*). Several reports recently showed that human *Vibrio* illnesses are increasing worldwide; these illnesses include fatal acute diarrheal diseases, such as cholera, gastroenteritis, wound infections, and septicemia (*1*,*4*). Fatalities associated with *Vibrio* infections are more common in persons who are immunocompromised or who have underlying diseases or syndromes, such as immune disorders, diabetes, and HIV/AIDS, than in persons without these conditions. Critically, *Vibrio* bacteria grow preferentially in warm (>15°C), low salinity (<25 parts per thousand NaCl) seawater (*4*,*5*). Warming of low-salinity marine environments is likely to support larger numbers of *Vibrio* populations and consequently increase the risk for vibriosis. In this regard, during the past 2 decades, reported infections have increased that have spread poleward and in areas not usually associated with these bacteria, including temperate and cold regions, such as the US Pacific Northwest (*6*,*9*), South America (*7*,*8*), and northern Europe (*4*,*5*). We describe a highly unusual instance of a large number of *Vibrio* infections reported in high-latitude coastal counties in northern Europe during summer 2014.

## Materials and Methods

During winter 2014 and into the early spring 2015, we became aware of an unusual number of reported *Vibrio* infections in northern Europe. Colleagues at the European Centre for Disease Control relayed the initial information to the Centre for Environment, Fisheries and Aquaculture Science (Weymouth, UK) and the University of Bath (Bath, UK). The information suggested that an unprecedented number of *Vibrio* infections had been observed in Sweden and Finland during summer 2014 and that many cases were reported in high-latitude coastal counties.

To scrutinize cases of infection, we took several approaches. We initially contacted the Public Health Agency of Sweden (Stockholm, Sweden) and the National Institute for Health and Welfare (Helsinki, Finland), as well as other northern Europe reference laboratories, in December 2014. Although vibriosis is not regionally notifiable in Europe, Finland and Sweden maintain national databases of *Vibrio* infections. In Finland, *V. cholerae* is a notifiable infection, and isolates from persons with suspected infections are submitted to the reference laboratory for confirmation, serotyping, and PCR testing for the cholera toxin gene (*ctx*). Also, other *Vibrio* species (e.g., *V. vulnificus*, *V. parahaemolyticus*) may be sent to the reference laboratory for subsequent species-level confirmation. In Sweden, diarrhea with CTX-producing *V. cholera* O1 or O139 is a notifiable disease, as is infection with other *Vibrio* species, including *V. cholerae* not producing CTX that causes wound infections, septicemia, enteritis, and otitis. Isolates of *V. cholerae* are sent to the Public Health Agency of Sweden for serotyping and confirmation of virulence factors, such as *ctx*, using appropriate molecular methods, such as PCR. 

For cases identified in 2014, the geographic location of each reported infection was established (e.g., town or city where the patient was treated). Where possible, information relevant to disease transmission, such as possible water-associated activities, also was gathered; however, for many cases, this information was not available. Basic epidemiologic data on each case, including patient sex and age, was subsequently collated, as was the site of bacterial isolation (e.g., wound, ear, blood). The date the case was reported to regional authorities was determined, and for a subset of cases, data on the onset of reported symptoms also were established. To assess recent trends regarding infections, we collated *Vibrio* cases identified in Finland and Sweden from 2005 onward and omitted from analysis cases we suspected of being foreign-acquired.

To assess the possible role of extreme weather events on the emergence and dynamics of *Vibrio* disease in Finland and Sweden, we analyzed the epidemiologic data alongside long-term sea surface temperature (SST) records (HadISST [Hadley Centre Sea Ice and Sea Surface Temperature dataset] and ERSST [Extended Reconstructed Sea Surface Temperature dataset, v3b from the US National Oceanic and Atmospheric Administration (NOAA)] [*4*]). We used satellite-derived data to scrutinize temperature conditions and changes in the Baltic Sea area using NOAA’s Optimum Interpolation v2 Daily SST Analysis dataset that integrates satellite SST data retrievals. NOAA data (baseline period of 30 years [1971–2000]) was used to determine anomalies from this dataset. We also scrutinized daily SST and SST anomaly retrieval data from 6 fixed positions in the Baltic Sea area, which included the transitional waters between southern Sweden and Denmark, the southeastern and mideastern Baltic coasts of Sweden, and Bay of Bothnia (northern Baltic) and southern coast of Finland. To assess the significance of climatologic data from summer 2014, we also used long-term oceanographic datasets to analyze SST. In situ SST was provided by the Finnish Meteorological Institute and was downloaded on November 14, 2014. We also used instrumental measurements of SST in coastal areas in the Baltic Sea area. We removed short-term fluctuations from the buoy data by applying a 1-hour wide median filter to the original dataset.

Statistical tests used to infer the relationship between maximum SST and annual *Vibrio* case occurrence were investigated by using a generalized linear model that assumed a quasi-Poisson error distribution (log link function) in R version 3.1.3 (http://www.R-project.org). We analyzed daily long-term SST and anomaly data (1981–2015) using a Welch *t* test (which enables analysis of the unbalanced size of the 2 datasets).

## Results

**Figure 1:**
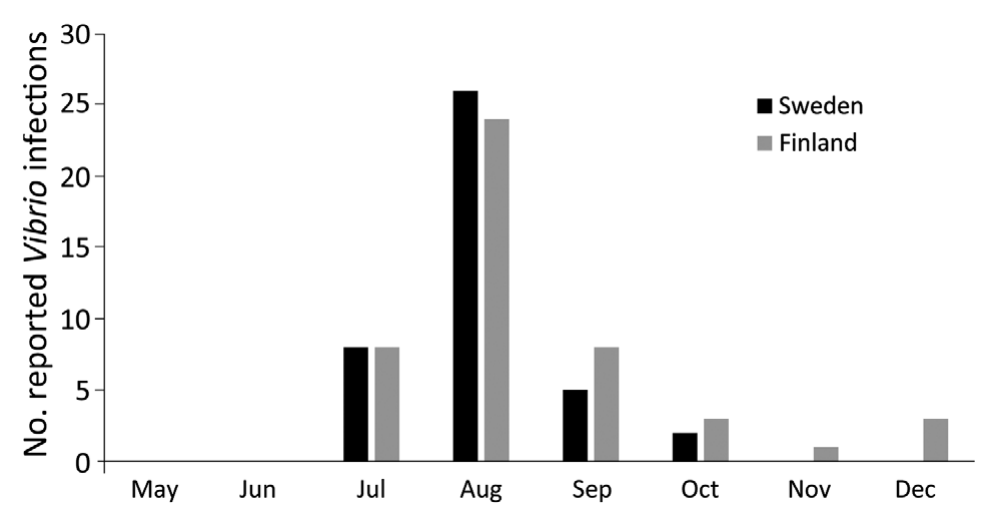
Monthly reported *Vibrio* infections in Sweden and Finland, May–December 2014. Beginning in July and increasing in August, reported infections spiked, corresponding with the heat wave in Scandinavia during that time.

A total of 89 *Vibrio* infections were reported in Sweden and Finland during the summer and autumn 2014, the largest yearly total number of cases, to our knowledge, identified in these countries. Infections were apparent across most Baltic coastal counties of Sweden and Finland. Numerous cases were reported at extreme subarctic regions, and as far north as >65°N, <100 miles (160 km) from the Arctic Circle. Reported infections began in July 2014 and peaked in August, before decreasing significantly in September (Figure 1). Infections were spread across persons of widely varying ages (range 3–93 years; median 36.2 years). In general, those infected were more commonly male (61 [67%] cases). One known fatality was noted: a *V. cholerae* non–O1/O139 infection reported in August 2014 from southern Sweden. Data on possible transmission was largely absent from the dataset from Finland; however, most cases in Sweden during 2014 occurred among persons who reported recreational exposure to seawater (e.g., the Baltic Sea) or lake water before infection (33 [78%] cases). Most (70 [77%]) infections were attributed to *V. cholerae* non–O1/O139; in 1 case, a *ctx*-negative O1 strain was reported. Other species reported were *V. alginolyticus* (3 cases), *V. parahaemolyticus* (4 cases), *V. vulnificus* (2 cases), *V. mimicus* (1 case), and unspecified *Vibrio* species (8 cases) (Table). Thirty-three (37%) infections were associated with ear or ear secretion isolations; however, for 17 (19%) of the 89 reported cases, *Vibrio* organisms were isolated directly from blood, suggesting more serious systemic disease progression.

**Table:** Relevant microbiologic data about *Vibrio* pathogens identified from reported cases, Finland and Sweden, 2014*

Species	Country of isolation (no. cases)
*V. cholerae* non-O1/O139	Finland (45), Sweden (25)
*V. cholerae*, O1, Inaba, El Tor, *ctx*–	Finland (1)
*V. alginolyticus*	Sweden (3)
*V. parahaemolyticus*	Sweden (4)
*V. vulnificus*	Sweden (2)
*V. mimicus*	Finland (1)
*Vibrio* spp.	Sweden (8)
*ctx–, negative for the cholera toxin gene.	

**Figure 2:**
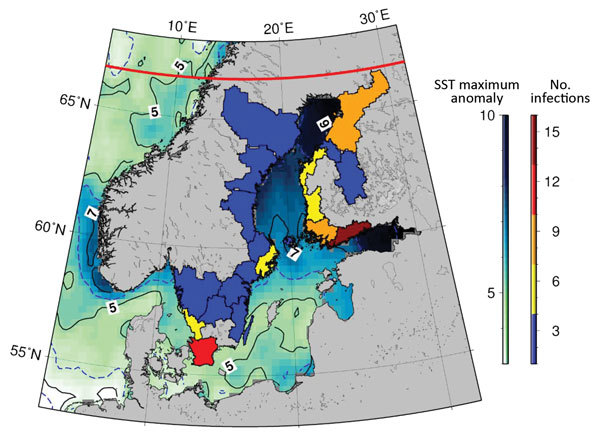
Location of reported *Vibrio* infections in coastal areas, Sweden and Finland, 2014. The number of infections coupled with the extreme SST anomaly, particularly in northern latitude areas, is particularly noteworthy. SST, sea surface temperature. Red line indicates the location of the Arctic Circle.

**Figure 3:**
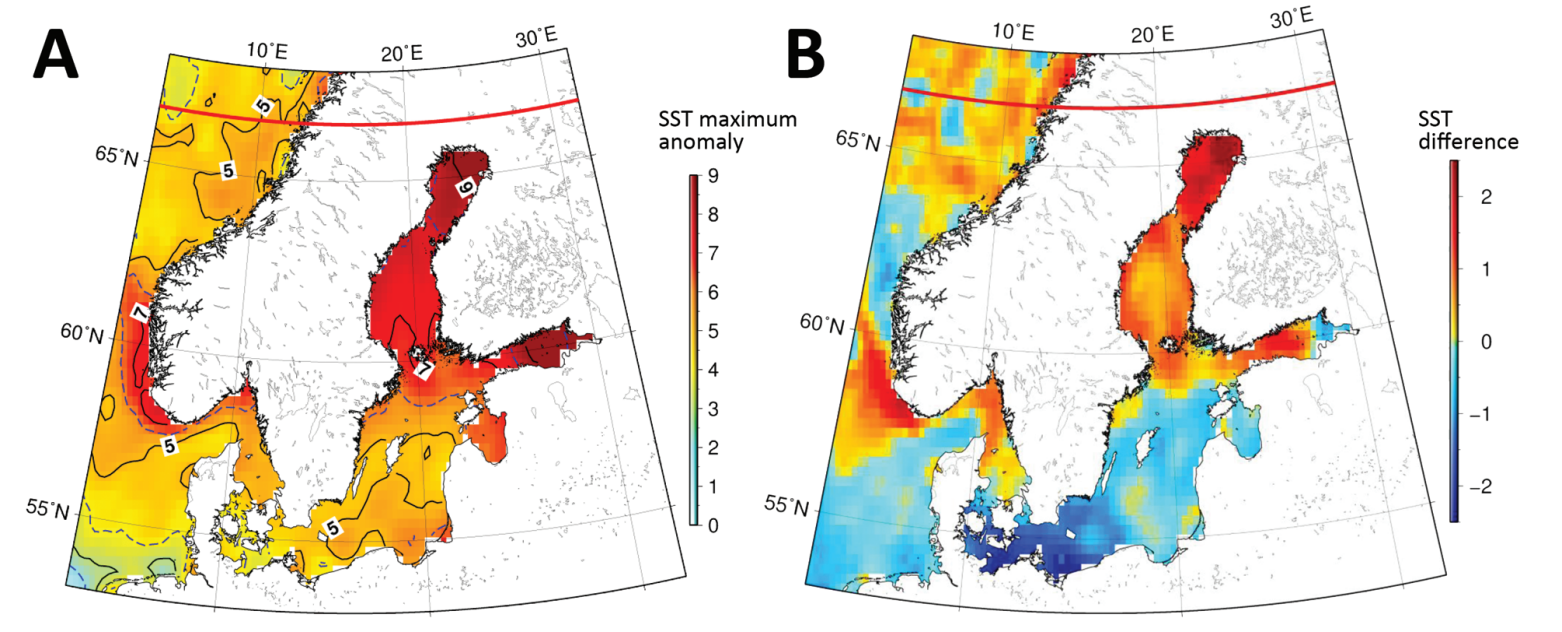
SST anomaly data for coastal areas of Sweden and Finland. A) Maximum SST anomalies during July and August 2014. The anomalies were substantially high throughout the region but especially in the northern Baltic Sea area. B) Differences between the maximum temperatures during 2014 and those during 1982–2013. SST, sea surface temperature.

The temporal and spatial distribution of reported cases corresponded closely with a highly anomalous heat wave in northern Finland and Sweden during July and August 2014, where SSTs in the northern Baltic exceeded all known long-term climatic and oceanographic records. A persistent and long-lasting period of high pressure occurred in northern Finland and Sweden beginning in May 2014, and this weather pattern persisted until mid-August. Concomitantly, SST in the Baltic Sea area was highly anomalous during July and August 2014; temperatures peaked toward the end of July. In some coastal regions, SSTs were ≈10°C higher than the long-term average, indicating the extreme severity of this anomaly (Figure 2). Across the northern Baltic Sea area, SSTs were several degrees Celsius warmer than had been reported since the early 1980s. SSTs across large swathes of the Baltic and the Gulf of Bothnia area, in particular, had SSTs >18°C for several weeks beginning in mid-July and ceasing in mid-August (Figure 3). SST’s reported in the Gulf of Bothnia at the end of July were the most extreme reported during 1981–2016, exceeding 21.7°C on July 29, 2014, and with several days of temperatures >20°C. The observed SST anomaly during this period was also the largest ever seen in this dataset, encompassing almost 13,000 data points, with an anomaly of 9.79°C on July 29, 2014. 

A statistical analysis between maximum SST and annual *Vibrio* cases using a generalized linear model showed that maximum SST explained a significant amount of the variability in cases (as determined by a significant reduction in the residual deviance from 120.55 to 42.16). The model predicted that, as the maximum SST increases, the number of annual number of cases also will increase significantly (β = 0.33002, SE = 0.08045, *t* = 4.102, p = 0.00343).

## Discussion

**Figure 4:**
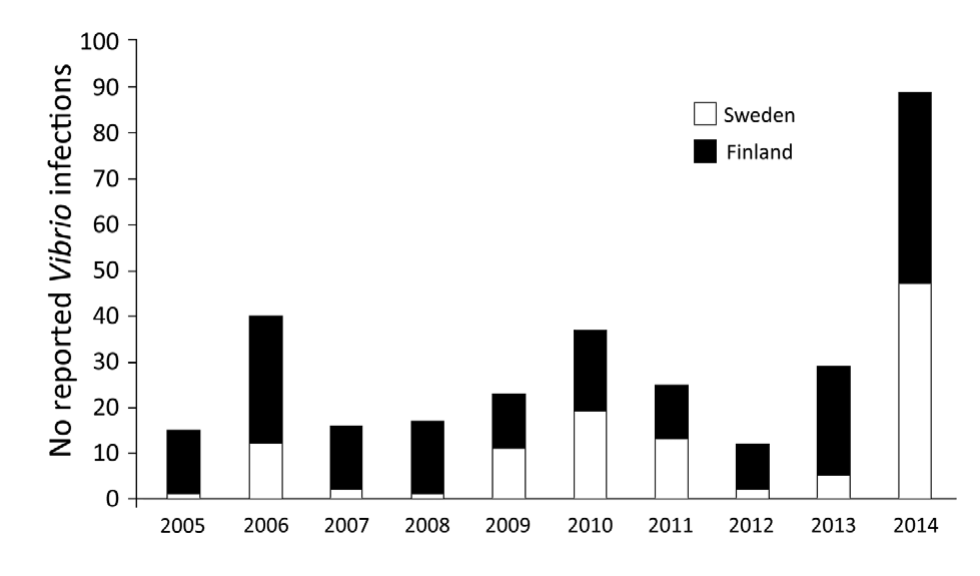
Total reported *Vibrio* infections in Finland and Sweden, 2005–2014. Foreign-acquired infections (where known) were omitted from the analyses. Epidemiologic data were gathered from public health agencies in Sweden and Finland (see Materials and Methods).

Domestically acquired *Vibrio* infections are rare in northern Europe, and the spike in recorded cases of vibriosis reported in this region is particularly noteworthy. The cases in 2014 are the largest yearly total of reported *Vibrio* infections in Sweden and Finland, more than double the number of reported cases than in other recent years (Figure 4). In Sweden, 2014 was the warmest year on record since recordkeeping began in 1860; in Finland, 2014 was the second-warmest year on record (*10*,*11*). Across Finland, 50 days of hot summer weather (temperatures >25°C) were recorded during May–August, which is 14 days more than the long-term average (*10*). The large number of reported infections corresponded closely with an intense and northerly SST anomaly, suggesting that these unusual oceanographic and climatic conditions drove this episode of waterborne disease. A subsequent quantitative and statistical analysis of SST data from this region revealed 3 further observations: 1) the peak SSTs in late July 2014 were the most intense observed in the Bay of Bothnia; 2) the anomaly is the most intense in almost 35 years of climate data (1981–2015); and 3) the likelihood of such an event occurring based on recent climate data (1981–2015) is highly unlikely—the 2014 maximum observed temperature was significantly higher than the maximum expected based on the data for other years, and based on the distribution of maximum temperatures observed, a temperature this much higher than the mean would be expected only in 0.78% of years (once every 128 years).

*Vibrio* species such as *V. cholerae* grow preferentially in low-salinity warm water, and recreational exposure to water, which appears to have been responsible for a sizeable proportion of these reported infections, also increases substantially during heat waves. That 2014 followed several other recent heat wave years (e.g., 1994, 1997, 2003, 2006, and 2010), during which recorded domestically acquired *Vibrio* cases spiked in northern Europe (*4*,*5*), is particularly noteworthy. Previous epidemiologic analysis regarding the emergence of *Vibrio* infections in the region (*5*) indicated that sustained SSTs >18°C were a notable risk factor, significantly increasing reported cases. The relation between maximum SST and annual *Vibrio* case occurrence analyzed by using generalized linear model–based methods demonstrated similarly to previous studies in the region (*4*) that maximum temperature correlates highly with risk, and cooler years (e.g., 2005, 2007, and 2012) indicate lower levels of reported infections than heat wave years (e.g., 2006, 2010, and 2014). In our study, the observation that a sizeable proportion of described cases were reported in subarctic latitudes (>65°N) and within 100 miles (160 km) of the Arctic Circle is striking. Ten *V. cholerae* infections were reported above 63°N, of which 6 cases were identified in the Oulo area (≈65°N). The cases recorded here are, to our knowledge, the most northerly reported instances of vibriosis documented, exceeding previous studies where cases have been reported at high latitudes, such as Alaska (*9*) and previously in northern Europe (*5*).

Disease data, such as those reported here, often are sporadic and usually grossly underreported. Likewise, a major limitation of our investigation was the lack of detailed trace-back epidemiologic data, which limits the assessment of exposure and subsequent risk. For many reported cases, data about prior exposure (e.g., specific information about the timing and location of recreational exposure to water) and subsequent routes of transmission were absent. However, almost without exception, cases from Finland and Sweden were reported in coastal rather than inland medical centers. Second, when prior transmission information was available from confirmed cases, most patients reported exposure to seawater in the days before symptom onset. These 2 factors, coupled with the striking climatic and oceanographic conditions during summer 2014, suggest that exposure to seawater was largely responsible for these episodes of disease emergence. The limitations underscore the need for a centralized system of surveillance and reporting. In the United States, the Centers for Disease Control and Prevention’s COVIS (Cholera and Other Vibrio Illness Surveillance) maintains a national database of vibriosis that contained detailed epidemiologic and transmission route information (*12*). A similar centralized reporting, monitoring, and surveillance system would greatly enhance risk assessment and risk management of vibriosis in Europe. Across the region, and with the exception of toxigenic *V. cholerae* infection, vibriosis is not a notifiable disease (*5*). Given that these rare waterborne infections appear to have emerged and increased in northern Europe recently (*13*) (e.g., 1994, 2006, 2014), this event underlies the need for clinicians to identify possible exposure to seawater. This event is particularly relevant for patients who have a history of conditions where progression of vibriosis to systemic infection is more likely, including diabetes, immune disorders, and liver dysfunction.

Climatic anomalies, such as the heat wave conditions during summer 2014 in northern Europe, appear to be responsible for restructuring the geographic distribution of waterborne infectious diseases and resulted in major and far reaching consequences for the identification, treatment, and management of these pathogens. The greater number and intensity of large heat wave events in northern Europe during the past 20 years or so (1994, 1997, 2003, 2006, 2010, 2014) further highlights the need for improved epidemiology and reporting, coupled with enhanced diagnostic capability in clinical settings to manage and ameliorate risk.
